# The Causation of Gastric Ulcer

**Published:** 1908-06-06

**Authors:** 


### Points in Surgery.

### THE CAUSATION OF GASTRIC ULCER.

Among the problems presented by modern surgery few are so fascinating as an investigation into the causation of gastric ulcer. Where so little is known most of the suggestions hitherto put forward have been frankly speculative; and none of them, with the exception of one which will be mentioned later, is supported by any observed facts or serious experiments. Most of them, in fact, are more interesting than convincing, and some of them demonstrate the extremes to which enthusiasm and imagination can drive a scientific mind.

Some of the suggestions are based upon the observation that gastric ulcer attacks a particular class of individual, the common type being, of course, unhealthy anæmic young women. The next step is to single out some habit or characteristic which distinguishes them, and to jump to the conclusion that that habit or characteristic is necessarily the cause of the gastric ulcer. It is as unsound as to argue that because all trees are green everything which is green must be a tree. Such methods have evolved the suggestion that the ulcer may be caused by compression of the stomach owing to tight-lacing.

Those who are familiar with the working of the out-patient department of a hospital will not need to be reminded of the very large number of anæmic young women who present themselves with symptoms of dyspepsia and chronic gastritis, and, in worse cases, with those of gastric ulcer. The dyspepsia and gastritis are primarily the outcome of injudicious diet coupled with an existence in which a sufficiency of fresh air cannot be obtained. Anæmia follows later. Now, is the anæmia the result of the conditions mentioned, or does it cause them? On the face of it the former is the more probable hypothesis. But it has been said that the anæmia leads to a lack of oxygen, which devitalises the stomach wall, and so allows an ulcer to form. But why should the lack of oxygen devitalise the stomach wall at one circumscribed spot, and even if it does so how exactly does it predispose to ulcer formation? Both questions remain unanswered.

Another hypothesis, not less vague, supposes that the ulcer is due to some interference with the processes which normally prevent auto-digestion of the gastric mucous membrane. We know that the stomach secretes an enzyme which is capable of digesting food which is composed of the same constituent parts as the stomach itself; and it is obvious that in the living animal there exists some mechanism by which the pepsin is prevented from exerting its activity upon the stomach which has produced it. We know no more than this; and to say that an ulcer is the result of some interference with this natural process is merely to cloak our ignorance. Besides, the same objection applies as in the former case—why only at one circumscribed spot? Further, such auto-digestion, if it could be proved to occur, might conceivably produce an acute perforation of the stomach wall, but it could hardly explain satisfactorily the chronic ulcer which is associated with marked thickening of the neighbouring peritoneum.

The suggestions so far discussed may be classed under one heading: they are speculative, and therefore unscientific. A more reasonable idea is that clotting may occur in the smaller vessels, supplying a circumscribed area, which is deprived of its blood supply, and consequently necroses. And here, again, the concomitant anæmia is brought into the argument, because it is regarded as a possible cause of the clotting. Anæmia admittedly predisposes to, if it does not actually cause, intra-vascular clotting; and in many cases the actual vessel affected appears to be the result of sheer caprice. In some cases, of course, the vessel is damaged or diseased beforehand. Thrombosis is more common, for instance, in varicose veins than in normal ones, but in others there appears to be no discoverable predisposing factor. And why should not the small vessels of the stomach be selected as much as any other?

But when one considers the varieties of gastric ulcer, and the wide differences, for instance, between the chronic and the acute forms, it becomes more or less manifest that the only explanation which covers all the ground is that they must be the result of a definite specific infection. If that be admitted the next question to be answered is, What is the organism responsible?

Much attention is being paid nowadays to the bacillus coli communis. This micro-organism has, as we know, certain physiological functions, and as long as it remains in due bounds it performs them, and all is well. But if it escapes from its normal habitat, it becomes at once a violent enemy. We recognise this in appendicitis. Here, as the result of hyperactivity on its own part or of inefficiency on the part of the structures which normally restrain it, it gives rise to the phenomena associated with the various forms of appendicitis. It is quite reasonable to assume that under certain similar conditions it may invade the stomach, and cause the same changes there. For there are obvious points of similarity between the two conditions. Thus the acute perforated gastric ulcer may be likened to the gangrenous perforated appendix, while the chronic ulcer resembles more or less the relapsing variety.

Further, the hypothesis does not rest on speculation merely. In the last few months experiments have been made in America on the result of feeding dogs with an emulsion of the coli bacillus. Under normal circumstances the dog has hardly ever been observed to suffer from a gastric ulcer. Stray dogs were collected in the streets of Philadelphia and kept under observation. An emulsion of bacillus coli was mixed with their food, and in the course of time a large percentage of them acquired a gastric ulcer.

The experiment may or may not be regarded as convincing, but it does, at any rate, indicate a valuable line of research, and one which will repay the time spent upon it; if only because any experimental work which increases our knowledge of the action of the bacillus coli under abnormal conditions or in abnormal situations, is likely to shed light on the unsolved problems of abdominal surgery.

